# Painful Realities: Navigating the Complexities of Head and Neck Cancer Pain

**DOI:** 10.1111/odi.15150

**Published:** 2024-10-07

**Authors:** Hayden F. Byrd, Zachary A. Kohutek

**Affiliations:** ^1^ Department of Radiation Oncology Vanderbilt University Medical Center Nashville Tennessee USA

**Keywords:** head and neck cancer, pain, quality of life

## Abstract

**Background:**

Head and neck cancer (HNC) and its treatments can cause significant pain, which can profoundly impact patients' quality of life and treatment outcomes. Understanding the full scope of HNC pain is essential for effective management and improved patient care.

**Objective:**

This review aims to comprehensively analyze the multifaceted nature of pain experienced by individuals with HNC, including its various etiologies and management strategies.

**Results:**

HNC pain can arise from tumor extent, treatment‐related toxicity, or comorbid conditions. The pathophysiology involves complex interactions between nociceptive, neuropathic, and inflammatory mechanisms. Optimal pain control requires a multimodal patient‐tailored approach utilizing both pharmacological and non‐pharmacological therapies.

**Conclusion:**

Enhancing our understanding of HNC pain and optimizing its management is imperative for improving the overall well‐being and treatment outcomes of affected individuals. Future research should focus on understanding detailed pain mechanisms, with the goal of developing personalized pain management strategies and exploring novel therapeutic targets. By implementing comprehensive approaches to HNC pain management, healthcare providers can better support patients through their cancer treatment journey.

## 1 Introduction

Pain is highly prevalent in head and neck cancer (HNC) patients and is a major cause of morbidity in this population. Frequent pain assessment is critical to provide optimal patient care (Fink 2000). Pain assessment in clinical practice is often performed using questions describing the location, intensity, and quality of pain. While pain can be classified based on sensorial characteristics, the experience of pain is more complex and personal, shaped not only by biological factors but also emotional, psychological, social, and cultural inputs (Scribante et al. 2023).

Pain can be broadly classified as either nociceptive, caused by inflammation and tissue damage, or neuropathic, caused by nerve damage. A third type of chronic pain known as nociplastic pain has also been recently described. This pain arises from dysfunction in the processing of pain signals within the nervous system, leading to the perception of pain without clear evidence of ongoing tissue or nerve damage or inflammation (Fitzcharles et al. 2021). This broad classification forms an important framework for understanding the mechanisms of pain and can help guide treatment recommendations.

For nociceptive pain, treatment should address the source of the pain, if possible. If pain is caused by lymphedema or skeletal muscle tightness, for instance, treatments should focus on relieving lymphatic congestion or improving the range of motion through physical therapy. These therapies should be supplemented with non‐steroidal anti‐inflammatory drugs (NSAIDs), acetaminophen, neuromodulatory agents, or opioids per the WHO pain ladder (WHO 2019). Neuropathic pain tends to respond better to neuromodulatory agents such as gabapentin, pregabalin, selective serotonin reuptake inhibitors, or tricyclic antidepressants. In contrast, nociplastic pain is most likely to be refractory to medical therapy and may require non‐pharmacologic treatments such as exercise therapy, cognitive behavioral therapy, or other multidisciplinary management approaches (Nijs et al. 2023).

Pain is frequently a combination of these subtypes, and over the course of HNC treatment, pain triggers and pain types may change (Khawaja et al. 2023). Understanding pain and its causes can ultimately guide clinicians in developing optimal strategies to manage pain. Below, we describe unique considerations at each phase of HNC management: from diagnosis to treatment and through survivorship.

## 2 Pain Assessment

Pain assessment is essential to allow for effective pain management. Because of the subjective and complex nature of pain, a variety of scales have been developed to more reproducibly and completely characterize pain. Several general pain reporting systems used frequently across medical disciplines include the Numerical Rating Scale (NRS), Verbal Rating Scale (VRS), and Visual Analog Scale (VAS), which rate pain on a numerical or categorical scale and can provide a rapid assessment of pain intensity (Table 1) (Kirkova et al. 2006). Other instruments such as the Pain Relief Scale (PRS) quantify the degree of pain improvement after intervention (Lee et al. 2015).

**TABLE 1:** Assessment tools for pain and psychological distress.

Assessment tool	Description
Simple pain assessment	
Numerical Rating Scale (NRS)	11‐point numerical rating scale to measure pain intensity from 0 (no pain) to 10 (worst pain imaginable)
Verbal Rating Scale (VRS)	Asks patients to categorize their pain intensity as “none,” “mild,” “moderate,” or “severe”
Visual Analogue Scale (VAS)	Measures pain severity by having patients mark a position along a continuous line between “no pain” and “worst pain imaginable”
Pain Relief Scale (PRS)	Measures changes in pain intensity over time by asking patients to rate their pain as “worse,” “the same,” or “complete relief” following an intervention
Detailed pain assessment	
Memorial Pain Assessment Card (MPAC)	Includes four separate scales to assess pain intensity, description, relief, and mood. It provides a multifaceted view of a patient's pain experience and is quick to use
Brief Pain Inventory (BPI)	Assesses pain “right now,” “average,” “least in the past 24 h,” and “worst in the past 24 h,” and evaluates how pain interferes with daily activities such as general activity, walking, mood, sleep, work, pleasures in life, and social activity. The BPI is brief, self‐administered, available in multiple languages, and can be completed remotely
McGill Pain Questionnaire (MPQ)	Assesses pain through a series of descriptive words, allowing patients to describe the quality and intensity of their pain. It provides a comprehensive assessment by categorizing pain into sensory, affective, and evaluative dimensions
PROMIS Pain Instruments (Behavior, Interference, Intensity, Quality Neuropathic Pain, Quality Nociceptive Pain)	Part of the Patient‐Reported Outcomes Measurement Information System, these instruments provide detailed insights into the characterization of pain and its functional interference on a patient's daily life and activities
Broad symptom assessment	
Edmonton Symptom Assessment System (ESAS)	Evaluates multiple symptoms, including pain, in cancer patients. Patients rate the severity of their symptoms on a scale from 0 to 10, making it a quick and easy tool for both patients and healthcare providers
EORTC QLQ‐C30 and QLQ‐H&N35	These are part of the European Organization for Research and Treatment of Cancer's quality of life questionnaire modules. The QLQ‐C30 assesses general cancer‐related symptoms and quality of life, including pain, while the QLQ‐H&N35 focuses specifically on head and neck cancer symptoms, providing a detailed assessment of pain in areas such as the mouth, jaw, and throat
M.D. Anderson Symptom Inventory—Head and Neck Module (MDASI‐HN)	Measures the severity of symptoms experienced by head and neck cancer patients and the impact of these symptoms on daily activities. It includes specific items related to pain, making it a comprehensive instrument for symptom burden assessment
Vanderbilt Head and Neck Symptom Score (VHNSS)	Assesses symptom burden of patients undergoing radiation or chemoradiation for HNC, including subscales for nutrition, pain, voice, swallow and mucous/dry mouth
Patient‐Reported Outcomes version of the Common Terminology Criteria for Adverse Events (PRO‐CTCAE)	Has a specific items set for head and neck cancer, designed to capture acute symptomatic toxicities during radiation therapy. It includes detailed questions on pain, allowing for precise monitoring of patient experiences
Psychological assessment	
Hospital Anxiety and Depression Scale (HADS)	Widely used in cancer settings to screen for anxiety and depression. It consists of 14 items, with 7 items each for anxiety (HADS‐A) and depression (HADS‐D). It helps identify psychological distress that may exacerbate pain perceptions
Patient Health Questionnaire‐9 (PHQ‐9)	This tool screens for depression and assesses the severity of depressive symptoms. It is a self‐administered questionnaire with 9 items reflecting DSM‐IV criteria for major depressive disorder, useful in cancer patients for identifying depression‐related pain
Profile of mood states (POMS)	The POMS assesses various mood states that could affect pain perception, including tension‐anxiety, depression‐dejection, anger‐hostility, vigor‐activity, fatigue‐inertia, and confusion‐bewilderment. It is helpful in evaluating the emotional aspects of pain in cancer patients
Brief symptom inventory (BSI)	Measures psychological distress and psychiatric disorders. It includes 53 items covering dimensions such as somatization, obsessive‐compulsiveness, interpersonal sensitivity, depression, anxiety, hostility, phobic anxiety, paranoid ideation, and psychoticism
Beck depression inventory (BDI)	The BDI is a self‐report questionnaire that assesses the presence and severity of depressive symptoms. It includes 21 items that describe specific symptoms or attitudes related to depression, providing insight into the psychological aspects of pain in cancer patients

Assessing intensity alone provides only a partial characterization of pain, however, and other scales can be used to better describe pain and its impact on patient well‐being. Among these is the Memorial Pain Assessment Card (MPAC), which provides a quick and basic description of pain while assessing pain intensity, quality of relief, and patient mood related to pain (Fishman et al. 1987). Other scales such as the Brief Pain Inventory and the McGill Pain Questionnaire utilize additional questions to more completely characterize patient pain and its impacts (Melzack 1975; Cleeland and Ryan 1994). The Patient‐Reported Outcomes Measurement Information System (PROMIS) also contains several unique instruments that provide a detailed characterization of pain quality, intensity, and effect on patients (Amtmann et al. 2010).

In the setting of HNC, pain is often accompanied by a wide range of other symptoms. More general symptom reporting systems utilized for HNC that contain pain symptom clusters include the EORTC QLQ‐H&N35, the MD Anderson Symptom Inventory Head and Neck Module (MDASI‐HN), and the Vanderbilt Head and Neck Symptom Survey (VHNSS) (Bjordal et al. 2000; Cleeland et al. 2000; Rosenthal et al. 2008; Ridner et al. 2018). For assessing symptomatic toxicities during chemotherapy or radiation treatments, the Common Terminology Criteria for Adverse Events (CTCAE) and the related Patient Reported Outcomes (PRO‐CTCAE) version can be used (Dueck et al. 2015).

Beyond physical pain, it is important to consider the interplay between pain and psychological distress. Several screening questionnaires have been developed to assess for depression, anxiety, and other psychiatric disorders. Patients reporting pain can be quickly screened for anxiety and depression using the Hospital Anxiety and Depression Scale (HADS) or the Patient Health Questionnaire 9 (PHQ‐9) (Zigmond and Snaith 1983; Kroenke, Spitzer, and Williams 2001). Those with signs of psychological distress can be screened using more in‐depth scales such as the Profile of Mood States (POMS), Brief Symptom Inventory (BSI), or Beck Depression Inventory (BDI) (Derogatis and Melisaratos 1983; Beck et al. 1996). Together, these tools can help provide a comprehensive understanding of an individual's pain, which is critical in order to help appropriately tailor interventions.

## 3 Pain at Diagnosis

Up to 80% of HNC patients have pain prior to any treatment (Everdingen et al. 2007). The presence of pain at diagnosis has been shown to be directly linked to poor quality of life, and it is often the symptom that causes patients to seek evaluation (Oliveira, Zeidler, von Podestá, et al. 2014). While the nature of pain varies widely between patients, neuropathic pain, which is often described as burning or “pins and needles” type pain, affects over half of patients at diagnosis (Cuffari et al. 2006; Salwey et al. 2020). Neuropathic pain may develop due to tumor growth encroaching on nerves or from direct perineural spread of a tumor, which in addition to pain can cause numbness and muscular denervation (Dankbaar et al. 2018). The site of the primary tumor is often the most painful location, but patients commonly experience neuropathic pain in more distant areas of the head and neck region as well (Ye et al. 2023). Referred otalgia, for example, can result from the involvement of numerous cranial nerve branches including branches of cervical nerves (C II and C III) and cranial nerves V, VII, IX, and X that course throughout the head and neck region (Norris and Koontz 2020).

The best way to manage pain at presentation is prompt initiation of tumor‐directed therapies. Prior to treatment, pain can be managed with drug therapies aimed at temporarily reducing pain, including acetaminophen, NSAIDs, and opioids. Gabapentin may be particularly effective in patients with dominant neuropathic pain and should be started at low doses and titrated as tolerated. Patients with significant edema may benefit from a short course of corticosteroids. Patients should be counseled that pain may temporarily worsen should they pursue a nonsurgical approach, as radiotherapy may cause transient inflammation that can temporarily aggravate pain responses.

## 4 Perioperative Pain

HNC patients often undergo complex surgeries that are accompanied by a high risk for complications and perioperative pain. Perioperative pain management is critical, not only for improving patient well‐being, but also for limiting hospital length of stay and associated healthcare costs. In 2017, an international group of experts published an Enhanced Recovery After Surgery (ERAS) protocol specifically focused on HNC patients, with the goal of expediting postoperative recovery in this high‐risk group (Dort et al. 2016). Other clinical practice guidelines have been developed to specifically optimize the management of postoperative pain, with the goal of identifying risk factors for opioid use disorder, counseling patients regarding pain expectations, and utilizing multimodal analgesia (MMA) to reduce opioid use (Anne et al. 2021). While these guidelines provide a general framework for patient care, it is also critical to understand that head and neck surgical patients are a heterogeneous group. Pain severity and duration may vary greatly between procedures, ranging from mild–moderate pain lasting 1–3 days for endoscopic procedures to moderate–severe pain lasting for more than 10 days in the case of tonsillectomy or larger procedures requiring flap reconstructions (Schaller, Peterson, and Bäckryd 2021). In the case of transoral robotic surgery, which has become a widespread treatment modality in the management of early‐stage oropharyngeal squamous cell carcinoma over the last decade, only a limited amount of systemically collected postoperative pain data is available (Larsen, Kehlet, and von Buchwald 2023).

Risk factors for increased pain in the postoperative setting are numerous, but few are consistent between studies. Some of the most consistently identified risk factors include elevated preoperative pain levels or opiate requirements. Patients may also be more prone to severe postoperative pain if they have baseline anxiety or psychological distress, high expectations of pain, or are more prone to catastrophizing (Suffeda et al. 2016). Current smoking and marijuana use have been suggested as risk factors in some studies (Anne et al. 2021). In addition, tumor and treatment‐related factors including tumor site, pathologic stage, and surgical complexity are known to be important predictors of postoperative pain (Sommer et al. 2009; Bianchini et al. 2016).

Before surgery, patients should be counseled regarding expectations about pain duration and severity, as this may reduce postsurgical opioid requirements (Wittekindt et al. 2012). Opiate pain medications should be used for the shortest duration and at the lowest dose necessary to control pain effectively. In addition to opioids, recent studies have demonstrated that MMA using alternative non‐opioid pharmacologic agents may reduce perioperative pain within the first 24 h (Sanders and Dawes 2016; Go et al. 2022a). In two systematic reviews on the topic of MMA use in HNC patients undergoing surgery, Go et al. found that 80% of high‐quality randomized controlled trials and retrospective studies reported reduced postoperative narcotic usage with MMA (Go et al. 2022b, 2022a). The most commonly employed medications in these MMA protocols were gabapentin/pregabalin, followed by NSAIDs, acetaminophen, and corticosteroids. Interestingly, while gabapentinoids were utilized in most protocols, the one randomized study of perioperative gabapentin alone failed to demonstrate a benefit (Townsend et al. 2018). There is no one single combination or dosing schedule that has shown a consistent benefit across trials, yet it is clear that nonopioid medications show promise in reducing perioperative pain in the HNC population.

## 5 Pain From Radiotherapy

Radiotherapy plays a critical role in the management of HNC. In modern treatment paradigms, radiation is utilized in the definitive or adjuvant setting to control disease or symptoms associated with malignant invasion with the benefit of organ preservation (Tupchong et al. 1991). Radiation is delivered across the skin, soft tissue, bones, glands, muscles, nerves, and mucous membranes of the head and neck, which can lead to a variety of acute and late toxicities. Many of the side effects experienced by patients during radiation are marked by complaints of pain. Ultimately, pain is one of the most critical and difficult symptoms the oncologist must manage during treatment.

Radiation‐induced oral mucositis is the major cause of radiation‐induced pain. The underlying mechanism for the development of mucositis has been studied thoroughly (Sonis 2004). Radiation can damage the basal epithelial layer, triggering mitotic death and apoptosis in mucosal epithelial cells that lead to ulceration and microvascular damage. In addition to pain related to mucosal changes, this process leads to a secondary inflammatory response and potential microbial colonization that can exacerbate pain (Sonis 2004). Mucositis pain typically develops after the second week of radiation with mucosal erythema developing and progressing from patchy to confluent. Patients develop ulceration of the mucosa which becomes diffuse. Pain gradually worsens, peaking during the fifth week of radiation and not substantially improving until several weeks post‐treatment. The pain associated with oral mucositis includes both oral cavity pain and odynophagia, which can lead to major challenges in maintaining patient nutrition (Iovoli et al. 2023).

Patients also often develop pain from radiation dermatitis during their treatment course. When radiation is used to target cervical chain lymphatics located just deep into the dermis, the skin may receive doses to the skin of greater than 50Gy. The development of radiation dermatitis mirrors that of mucositis, with the depletion of replicative cells in the dermis leading to significant cytokine‐mediated inflammation (Yang et al. 2020). Early in the clinical course, dermatitis can manifest as pruritic, dry skin which progresses to patchy followed by confluent erythema. Dry and moist desquamation can occur toward the end of or in the weeks following radiotherapy, which poses an infection risk for the patient. Radiation dermatitis pain, like mucositis, is progressive, multifactorial, and attributed primarily to inflammatory responses triggered by DNA damage (Yang et al. 2020).

### 5.1 Patient‐Related Risk Factors

While all HNC patients are at risk for developing pain during radiotherapy, numerous factors have been identified that increase the likelihood of more severe pain. Among these predisposing factors are modifiable risks such as poor oral hygiene, tobacco use, or alcohol abuse. The etiology of many HNCs can be attributed to prolonged substance use with tobacco and alcohol (Barsouk et al. 2023). Despite this, many patients who are smoking at the time of diagnosis continue to smoke through treatment (Do et al. 2004). While nicotine has some known analgesic properties, evidence points toward patients who are current smokers during treatment experiencing higher self‐reported pain levels than their never smoker or even their former smoker counterparts (Weingarten et al. 2008; Logan et al. 2010). Furthermore, a history of or ongoing substance use disorder can significantly complicate the management of pain during treatment. While the etiology of worsened pain is unclear, smoking cessation is strongly encouraged—not only for improvement in tissue repair and prevention of further carcinogenesis but also to decrease pain in these patients.

In addition, patient demographic factors and comorbid medical conditions are known to impact risk for more severe pain during treatment. Perhaps the comorbidity most closely linked to pain is depression. Half of HNC patients experience some form of depression during their treatment course (Lydiatt, Moran, and Burke 2009; Yadav et al. 2019), often triggered by a combination of their diagnosis, the protracted and potentially disfiguring therapies, and neurocognitive changes brought about by cancer and its treatment (Lydiatt, Moran, and Burke 2009). An emphasis is growing in assessing and aggressively treating patients with depression during HNC treatment as these patients typically have higher analgesic requirements and are more sensitive to somatic symptoms (Kroenke et al. 2010). A component of the pain experienced by these patients, in addition to the nociceptive pain described above, will be neuropathic/nociplastic in nature requiring neuromodulatory agents or non‐pharmacologic management (Nijs et al. 2023). Management of depression has also been shown to help cessation of comorbid conditions contributing to pain including tobacco and alcohol use (Duffy et al. 2006). Ultimately, appropriate management of depression can positively impact clinical outcomes (Huang et al. 2022).

### 5.2 Treatment‐Related Risk Factors

The nature and intensity of treatment also impact pain. When radio‐sensitizing chemotherapy is utilized, for instance, it can augment radiation‐induced pain and can cause pain from peripheral neuropathy (Group et al. 1991; Weber et al. 2003). Radiation dose and target volume can also greatly impact treatment‐related toxicity. With the development of intensity‐modulated radiotherapy, the dose can conform to tumor targets while minimizing the dose delivered to organs at risk, resulting in reduced acute and late effects of radiation including pain (Lee et al. 2007). The use of image guidance has also become commonplace, allowing for tighter margins and reduced treatment volumes, thus further reducing side effects (Navran et al. 2019). Investigations are ongoing to assess the value of new technologies including intensity‐modulated proton therapy and daily adaptive radiotherapy in further reducing dose to normal tissues (Kim et al. 2018; Taku et al. 2021; Delaby et al. 2023).

In addition to these technological advances, strides have been made in reducing radiation treatment volumes and radiation doses. For some time, it has been known that select subsets of patients with well‐lateralized disease may be spared elective radiation to the contralateral neck without increasing the risk for cancer recurrence (Taku et al. 2022). Such normal tissue‐sparing results in reduced pain and improved global quality of life (McDowell et al. 2020). More recently, treatment de‐escalation efforts have focused on HPV‐associated oropharyngeal cancers which display a more favorable response to treatment. Strategies have centered around reducing radiation dose, treatment volumes, or the use of chemotherapy (Kang et al. 2023). Other ongoing trials are aiming to further de‐intensify treatments in subsets of patients based on favorable cytogenetics, tumor hypoxia, or circulating tumor DNA (ctDNA) (Chera et al. 2020; Riaz et al. 2021).

Regardless of the exact treatment modality used, any patient undergoing treatment with radiation or chemotherapy is at increased risk for opportunistic infections such as oral candidiasis or herpes simplex virus ulcers. These can present during treatment and worsen oral pain and dysphagia (Pispero et al. 2022). Many bacterial species constituting the oral flora can become pathogenic in the setting of immunosuppression (ISOO et al. 2010). Data also suggest a contribution of skin flora to radiation dermatitis (Kost et al. 2023). Infection within the treatment field further propagates a local inflammatory state worsening the pain already experienced by these patients due to mucositis dermatitis and tumor burden.

### 5.3 Recommendations for the Prevention and Management of Acute Pain

It is important to manage uncontrolled pain, as it not only worsens patient quality of life during treatment but also leads to chronic swallowing dysfunction and suboptimal oncologic outcomes. Treatment‐induced pain can cause an inability to maintain oral intake, which can lead to the need for enteral feeding to maintain patient weight (Iovoli et al. 2023). Pain control is also essential to allow patients to maintain swallow function during radiation, as decreased swallow effort during treatment can translate into fibrosis and atrophy in the long term. In some patients, even if swallowing can be maintained in the short term, chronic pain can lead to the development of altered swallowing and long‐term feeding tube dependence (Bossola, Antocicco, and Pepe 2022). In addition, pain or the treatment of pain may require hospitalization, which itself can lead to missed radiation treatments, a lengthened treatment course, and reduced rates of cure (Mazul et al. 2020).

The management of acute pain during the treatment of HNC with radiation is challenging and is an area of active research. Addressing treatment‐associated pain management begins prior to treatment when assessing the patient during consultation for pre‐treatment pain, psychiatric concerns such as anxiety/depression, and social determinants like support and resources are crucial to adequately address prior to treatment initiation. Thorough patient and family counseling and education has also been shown to be an effective prevention strategy for pain (D'Souza et al. 2013). This often involves the multidisciplinary efforts of oncologists, psychiatrists/palliative care, and nutrition/speech therapists.

Early during radiation, sodium bicarbonate rinses can help soothe inflamed mucosa and should be continued throughout to help reduce the risk of infection. Best oral care is often supported with the addition of topical anesthetic solutions of doxepin or lidocaine‐based mouthwash to provide temporary relief of pain before meals (Sio et al. 2019). Pain typically becomes more severe over the course of treatment, often requiring systemic opioid pain medications. Data support early induction of opioids in addition to the continuation of non‐opioid analgesics as tolerated (Takase et al. 2011).

Updated guidelines from the Multinational Association for Supportive Care in Cancer (MASCC) also recommend consideration of 0.2% topical morphine rinse for mucositis treatment (Blanchard et al. 2022). Pharmacologic management of pain requires careful discussions between the care team and the patient. While each treatment course bears its own challenges, it is common for patients to require immediate release and long‐acting opioid analgesics by the end of treatment. Unfortunately, mucositis pain is often resistant to opiate pain medications, and despite aggressive pain management patients may require enteral feeding to maintain nutritional requirements throughout treatment.

In contrast to this reactive strategy of pain management, numerous therapies have been tested in the preventative setting with the goal of delaying the onset of or reducing the severity of mucositis‐related pain. The use of prophylactic gabapentin to reduce oral mucositis pain has gained particular interest in recent years, yet it remains controversial (Ad et al. 2010). In the only placebo‐controlled double‐blind study on this topic, Cook et al. found no statistical benefit to gabapentin in reducing Patient‐Reported Oral Mucositis Symptom (PROMS) scores (Cook et al. 2022). In contrast, Smith et al. found that prophylactic gabapentin during radiation resulted in a significant reduction in pain as measured by VHNSS 2.0 pain subscale scores (Smith et al. 2020), perhaps due to the different clinical endpoint that focused exclusively on pain. The ideal dosing strategy for prophylactic gabapentin remains unclear. Smith et al. used a dosing regimen of 100 mg thrice daily (TID) in Week 1 of treatment increased to 300 mg TID in Week 2, and up to 600 mg TID in Week 3 and 900 mg TID in Week 4 as tolerated. While an unplanned secondary analysis of two prospective clinical trials conducted by Ma et al. found that higher doses of gabapentin (3600 mg daily) were more effective than lower doses (900 or 2700 mg daily) in delaying opiate use, most patients in Smith et al. did not increase beyond 300 mg TID dosing, suggesting that high doses may not be needed for the pain‐reducing effect of gabapentin (Smith et al. 2020; Ma et al. 2022). The potential effects of prophylactic gabapentin may go beyond pain. Studies have demonstrated improved swallow function and reduced systemic symptoms after chemoradiation (Starmer et al. 2014; Smith et al. 2020). Trials are also ongoing to determine whether gabapentin may synergize with other neuromodulatory agents such as ketamine in the prophylactic setting (NCT05156060).

In addition to therapies aimed at reducing mucositis pain, a wide range of treatments have been tested with the goal of preventing the development of mucositis itself. The MASCC panel has recommended consideration of the anti‐inflammatory agent benzydamine for mucositis prevention. The first randomized placebo‐controlled trial investigating the use of benzydamine oral rinses was published 35 years ago, with Epstein et al. reporting a reduction in mucositis among 25 patients receiving benzydamine compared with 18 patients receiving placebo during fractionated radiation to 45–60 Gy over 3–5 weeks (Epstein et al. 1989). Since that initial study, numerous additional randomized controlled trials have demonstrated a benefit to benzydamine. A large multicenter placebo‐controlled study showed that a 0.15% benzydamine oral rinse used 4–8 times daily during and up to 2 weeks following radiation reduced oral mucositis in patients receiving up to 50 Gy of radiation alone (Epstein et al. 2001). Others have demonstrated that patients undergoing chemoradiation and higher doses of radiation also derive a benefit from benzydamine rinses (Kazemian et al. 2009; Rastogi et al. 2017). The most recent MASCC guidelines have also added a recommendation for oral glutamine, which is thought to act by limiting free radical damage and decreasing inflammatory cytokines in the oral milieu, based on 2 RCTs demonstrating oral glutamine at a dose of 10–30 mg daily during chemoradiation may prevent OM. (Blanchard et al. 2022). Probiotics have also been utilized to reduce the severity of mucositis, highlighting the importance of oral flora in the development of mucositis (Xia et al. 2021; Peng et al. 2024).

Intraoral photobiomodulation, or the use of low‐level energy light therapy to stimulate cellular regeneration and reduce inflammation, has also been recommended by the MASCC. While this therapy is promising, variations in exact protocols used with respect to parameters such as wavelength and fluence, as well as limited accessibility still limit its use. There is also controversy regarding potential long‐term effects including the possibility of PBM‐induced carcinogenesis, which will require further study (Zecha et al. 2016). A randomized trial has also demonstrated the efficacy of facial massages in reducing the onset and severity of mucositis (Yang et al. 2021). Mucositis may be complicated by oral candidiasis, which should be assessed regularly during treatment. If candidiasis is suspected on clinical exam, patients should be treated with a triazole antifungal or nystatin oral liquid.

For dermatitis, patients are encouraged to apply generous amounts of water and petroleum‐based lotions to involved sites daily to assist with reducing pain and preventing early desquamation. As dermatitis and associated pain progress, silver‐based creams serve to prevent infection. More recently, mometasone furoate has been proven in randomized trials to improve pain and itching‐associated dermatitis below 60Gy compared to controls (Liao et al. 2019).

## 6 Pain in Survivorship

As described earlier, the peak of acute pain takes place at the end of treatment. Following treatment, pain is expected to gradually improve over the following 3–6 months (Lou et al. 2021). Recovery from chemoradiation can follow a range of trajectories, however, with some patients recovering quickly and others having prolonged and incomplete symptom resolution (McDowell et al. 2023). The transformation into chronic pain can be marked by a more generalized, rather than focal, pain. Unfortunately, a significant portion of patients progress to the chronic subtype with approximately 40% requiring pain medication a year out from treatment (Lou et al. 2021).

### 6.1 Predictors of Chronic Pain

Chronic pain can be associated with tumor recurrence but frequently also develops in cancer survivors. While it can be difficult to predict whether a given patient will develop chronic pain after treatment, some factors have been shown to be associated with pain in HNC survivors. These include younger age, oral cavity location, tobacco use, presence of comorbidities, history of neck dissection, and presence of xerostomia (Scharpf et al. 2009; Shuman et al. 2012; Cramer, Johnson, and Nilsen 2018; Bolnykh et al. 2024). HNC patients with chronic pain are also more prone to have co‐occurring depression, anxiety, fatigue, cognitive impairment, and sleep disturbance. This neuropsychological symptom cluster is quite common, with over 60% of patients experiencing at least moderate symptoms in two domains at some point during their disease course. These symptoms tend to peak at the end of treatment, but over 40% have a persistence of symptoms at 1 year after treatment (Barandouzi et al. 2023). While the mechanisms underlying this phenomenon are not fully known, neuroimmune mechanisms have been implicated, with chronic pain and other neuropsychological symptoms being linked to unresolved chronic inflammation in the peripheral and central nervous system (Walker et al. 2014). HNC patients are known to have increased peripheral inflammation both from tumor and from treatment, and inflammatory cytokine levels have been linked to the prevalence of chronic pain in these patients (Oliveira, Zeidler, von Lamas, et al. 2014; Barandouzi et al. 2023). As of yet such predictors have not been used clinically, but in the future they have the potential to identify high‐risk patients and inform clinical management.

### 6.2 Causes of Chronic Pain

One of the most frequent causes of chronic pain is musculoskeletal impairment, which can result from fibrosis, nerve damage, or muscle injury from either surgical or non‐surgical management. In patients undergoing surgery, neck dissection can lead to the development of neck and shoulder pain, which can evolve into shoulder stiffness and capsulitis. Neck and shoulder disability after neck dissection can be caused by multiple mechanisms. Post‐surgical scarring may lead to restricted neck mobility, nerve damage can cause weakness of muscles such as the trapezius, and manipulation of the sternocleidomastoid muscle can cause decreased neck strength. There is a direct relationship between reduced musculoskeletal function and increased pain in these patients (Gane, O'Leary, et al, 2017). Preservation of the spinal accessory nerve can reduce the risk of shoulder hypomobility and pain, although it does not eliminate the risk (Terrell et al. 2000; Gane et al. 2017). Neck dissection can also predispose patients to develop myofascial pain syndrome, a condition characterized by deep and intense muscular pain at trigger points within tense bands of muscle (Cardoso et al. 2015).

Non‐surgical treatment has also been linked to high rates of neck disability, and patients undergoing adjuvant radiation after surgery are at markedly increased risk for musculoskeletal impairment (Nilsen et al. 2020). Radiation is known to stimulate a progressive fibrotic reaction in tissue that is proportional to the dose delivered and volume treated. Radiation fibrosis can affect any number of tissues including muscles, bones, tendons, ligaments, and nerves ‐ all of which can lead to impaired musculoskeletal function and pain. Early in its development, fibrosis can restrict motion or cause painful muscular contractures or spasms. Over time, fibrosis can progress to tissue atrophy, which can cause such conditions as “dropped head syndrome” resulting from neck extensor motor weakness that can lead to poor posture and chronic musculoskeletal pain.

In addition to musculoskeletal pain at the site of cancer, patients undergoing myofascial reconstruction requiring placement of a flap may develop morbidity and pain at the site of flap harvest (Archibald, Stanek, and Hamlar 2022). Of those patients undergoing reconstruction with an anterolateral thigh flap, for instance, 27% required treatment of donor site pain from 1 to 2 years postoperatively (Shen et al. 2022). Patients undergoing a radial forearm free flap have a reported 23% incidence of late donor site pain (Bruin et al. 2022). In addition, when the radial forearm free flap is harvested, there is the risk of damage to the sensory branch of the radial nerve which can cause accompanying hypoesthesia and permanent sensory changes. Other sites such as the fibular free flap have more limited data, but regardless of the donor site, it is important to be mindful of pain at that site as a possible consequence of surgical management (Hatchell et al. 2021).

Radiation can also cause chronic pain by directly damaging bones or nerves. High‐dose radiation to the jaw can cause mucosal breakdown resulting in bone exposure, devascularization of bone, and painful osteonecrosis (Singh et al. 2022). Chronic jaw pain can also develop from infection of surrounding tissues, from pathologic fracture, or from disruption of the inferior alveolar nerve (Hiraoka et al. 2018). Modern radiation techniques and dose limits have reduced the incidence of nerve damage, but even so, radiation can cause peripheral neuropathies including brachial plexopathy that can lead to chronic shoulder and arm pain (Chen et al. 2012).

A proportion of patients develop pain without a clear nociceptive cause, instead developing a phenotype of widespread pain. Underlying this phenotype is the process of central sensitization—a maladaptive pain response in which amplification of pain signaling occurs in the CNS leading to exaggerated pain responses to sensory stimuli. This concept of central sensitization, in which pain hypersensitivity results from maladaptive amplification of pain signaling in the CNS, is a major mechanism underlying nociplastic and occasionally chronic neuropathic pain (Nijs, Malfliet, and Nishigami 2023). In other conditions associated with central sensitization, including fibromyalgia and irritable bowel disease, pain is often associated with neuropsychologic symptoms such as those discussed earlier in this section. It remains unclear to what extent central sensitization contributes to chronic pain in HNC survivors, but this remains an area of active ongoing research.

### 6.3 Considerations in Managing Chronic Pain

The management of chronic pain should be multifaceted. Multiple guidelines have been developed to guide titration and selection of opioid medications, but a comprehensive pain management strategy should also employ non‐opioid medications and non‐pharmacologic strategies when indicated (Bossi et al. 2020). If patients develop musculoskeletal complications, in addition to opiate analgesics, they should be referred for specialized physical therapy and considered for NSAIDs. In patients with pain related to radiation fibrosis, physical therapy, and neck stretching exercises are often recommended to help release fibrotic tissues and improve the range of motion. In a meta‐analysis of exercise‐based rehabilitation in HNC, post‐surgical patients were seen to have improved pain levels after exercise intervention (Pérez et al. 2023). Although a clear benefit was not seen in patients undergoing chemoradiation, only two studies were available for analysis. Contractures or spasms can also be supplemented with antispasmodic medications or periodic focal injections of botulinum toxin. Botulinum toxin can be effective in treating cervical dystonia, post‐treatment muscular contracture, and trismus, and it has been shown to be particularly effective in improving masticator spasm and related pain after radiation (Dana et al. 2007; Stubblefield et al. 2008; Bach et al. 2012).

Musculoskeletal pain in HNC survivors has also been demonstrated to benefit from targeted yoga and acupuncture (Pfister et al. 2010; Adair et al. 2018). Pain should be managed by a multidisciplinary team and an individualized survivorship care plan including management of pain should be developed for each patient.

## 7 Conclusions and Future Directions

In recent years, as human papilloma virus‐associated cancers have become more common, the median age of HNC patients has decreased, and patient survival has increased. As patients continue to live longer after treatment, pain management and prevention among survivors will become even more critical. Efforts to de‐escalate treatment hold promise in reducing acute toxicity by limiting tissue damage. In parallel with these efforts, however, it will be important to continue to characterize the pathogenesis of pain triggers, with the goal of identifying treatments that reduce the incidence and severity of treatment‐related side effects or the pain they cause, regardless of the intensity of treatment delivered.

In addition to reducing acute pain triggers, it will be necessary to better understand the mechanisms by which acute pain transforms into chronic pain. While the development of nociplastic pain and central sensitization has been linked to chronic inflammation, it remains unclear to what extent an aberrant response to acute inflammation or an inability to resolve inflammation is to blame for chronic pain in this population. It is also not known to what extent genetic and epigenetic factors may alter a patient's ability to return to homeostasis after cancer treatment. By more precisely classifying chronic pain in these patients, it may be possible to attain a better understanding of the patient variables and biologic mechanisms that promote long‐term cancer pain, as well as which risk factors can be modified to help prevent a transition to chronic pain.

As we better understand the pathophysiology of pain in HNC patients, novel therapeutic targets are emerging to treat orofacial pain (Ji 2022; Raman et al. 2023). In addition, there is extensive research ongoing that aims to uncover how best to utilize existing non‐opioid pain medications. We have learned that pain is heterogeneous, and treatment must be tailored toward each individual patient's pain characteristics. With drug therapies that target a wider range of pain pathways, there will be the potential to achieve patient‐specific treatment. By implementing comprehensive and novel approaches to HNC pain management in the future, there is hope that healthcare providers can better support patients through treatment and patients will have improved long‐term quality of life after HNC treatment.

## Author Contributions

**Hayden F. Byrd:** writing – original draft, writing – review and editing. **Zachary A. Kohutek:** writing – original draft, writing – review and editing.

## Conflicts of Interest

The authors declare no conflicts of interest.
